# Predicting cellular adaptation proteins dependent on eIF2α regulation under stress conditions: Physiological and pathophysiological implications in neuronal function

**DOI:** 10.1016/j.csbj.2025.07.015

**Published:** 2025-07-12

**Authors:** Víctor Herrera-Fernández, Hugo Fanlo-Ucar, Patrick Gohl, Melisa Ece Zeylan, Simge Senyuz, Ozlem Keskin, Attila Gursoy, Rubén Vicente, Baldo Oliva, Francisco J. Muñoz

**Affiliations:** a Laboratory of Molecular Physiology, Department of Medicine and Life Sciences, Faculty of Medicine and Life Sciences, Universitat Pompeu Fabra, Barcelona 08003, Spain; b Center for Pathobiochemistry and Genetics, Medical University of Vienna, Vienna 1090, Austria; c Laboratory of Structural Bioinformatics (GRIB), Department of Medicine and Life Sciences, Faculty of Medicine and Life Sciences, Universitat Pompeu Fabra, Barcelona 08003, Spain; d Computational Sciences and Engineering, Koç University, Istanbul 34450, Türkiye; e College of Engineering, Koç University, Istanbul 34450, Türkiye

**Keywords:** eIF2α, Integrated stress response, Cell translation, Synaptoplasticity, Neurodegeneration, SLC30A4

## Abstract

Understanding the intricate mechanisms governing gene expression regulation is crucial for deciphering neuronal responses to cellular stress at both the physiological (i.e., synaptogenesis) and pathophysiological (i.e., neurodegenerative diseases) levels. These rapid adaptive changes depend on the translation of specific proteins with specialized 5′ untranslated regions (5′UTRs), triggered by the phosphorylation of eukaryotic initiation factor 2 alpha (eIF2α), while normal cellular translation remains largely inhibited. This study aims to provide a useful tool to identify mRNAs susceptible to regulation by p-eIF2α. We compiled a database of 5′UTRs using Ensembl canonical transcripts from the GRCh38.p14 genome build. Ensembl IDs were used to extract coding sequences and cDNA via the REST API, and 5′UTR regions were identified. We applied translation efficiency-based filters to existing databases of p-eIF2α-dependent translation to obtain reliable training and testing datasets. A multiple logistic regression (MLR) model—using 5′UTR length, GC content, upstream open reading frames (uORFs), and the features of Atf4 as a reference—predicted scores for p-eIF2α-driven translation. Gene Ontology (GO) enrichment analysis identified significant biological processes, molecular functions, and cellular components involved. An interactome analysis using STRING-db highlighted pathways related to synaptoplasticity (physiological stress) and Alzheimer’s disease (pathophysiological stress). *In vitro* luciferase assays validated *SLC30A4* as a novel p-eIF2α-regulated transcript, uncovering the role of eIF2α regulation in zinc homeostasis and neurodegeneration. These findings underscore the importance of translational control mechanisms in memory formation and disease pathogenesis, contributing to the identification of potential therapeutic targets to mitigate pathological outcomes.

## Highlights

● MLR modeling predicts p-eIF2α-dependent effectors with high specificity based on intrinsic features of their 5′ UTRs.
● GO term and interactome analyses highlight the predicted ISR effectors' relevance in synaptoplasticity and neurodegeneration.
● *In vitro* validation confirms the model’s predictive performance and identifies SLC30A4 as a novel ISR effector.

## Graphical Abstract

**Figure:**
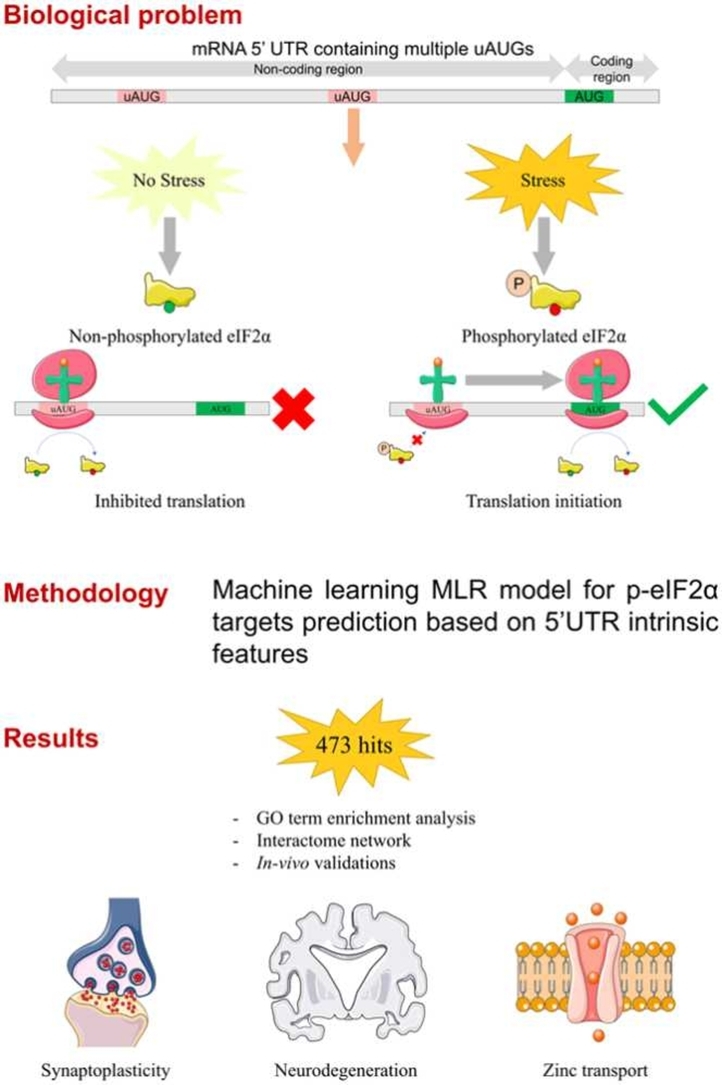

## 1 Introduction

Gene expression begins with the transcription of DNA into mRNA and concludes with protein translation, following a conventional pattern for most proteins. However, unlike transcriptional regulation, direct translational control of pre-existing mRNAs enables swift adjustments in cellular protein levels. This dynamic mechanism facilitates rapid adaptation to various stressors or nutrient deficiencies, which is crucial for maintaining cellular homeostasis in challenging environments [1].

**Fig. 1:**
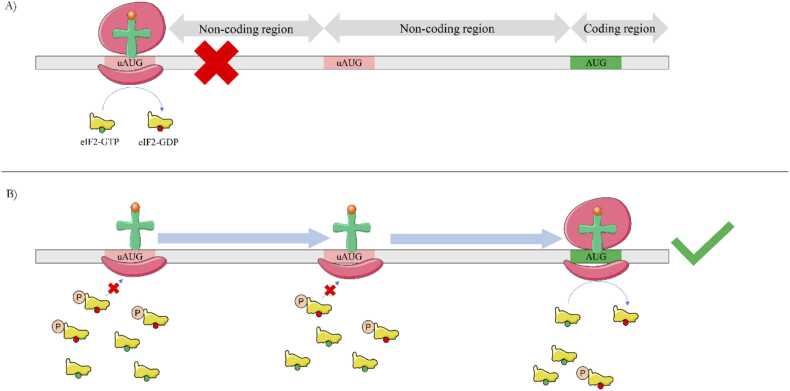
The p-eIF2α-dependent translational control mechanism. A) Under normal conditions, scanning and initiation of mRNAs with three or multiple AUGs cannot occur. The scanning ribosome encounters an upstream ORF, triggering GTP hydrolysis by eIF2-GTP (the active form of eIF2, green), which leads to the release of eIF2-GDP (the inactive form of eIF2, red). This results in translation initiation at a non-coding region and inhibits protein translation of those specific transcripts. B) When eIF2α is phosphorylated under stress conditions, the availability of eIF2-GTP is reduced. This leads to a leaky scanning mechanism, increasing the likelihood of eIF2-GTP binding to the correct ORF to initiate protein translation.

The initiation phase, the most rate-limiting step of protein translation [2], is under the control of eIF2. Initially, eIF2 forms the ternary complex, bringing methionyl t-RNA to the 40S subunit of the ribosome, enabling its scanning mechanism for the AUG start codon [1], [3], as shown in Fig. 1. eIF2 is a heterotrimer composed of the alpha, beta and gamma subunits. While the beta and gamma subunits are in charge of binding other necessary initiation factors, RNA, and GTP, alpha subunit has a cellular stress-dependent regulatory role. Phosphorylation of eIF2α at serine 51 represses the translation of most mRNAs, but paradoxically enhances the translation of mRNAs containing multiple upstream initiation codons (uAUGs; Fig. 1) and internal ribosome entry sites [1], [4]. Three main features in the 5′UTR define the mRNAs whose translation is enhanced by eIF2α phosphorylation: long length, multiple uAUGs, and high GC content [5], [6]. Many of these mRNAs encode proteins required under specific conditions, such as harmful situations [7]. eIF2α phosphorylation also occurs during synaptogenesis, a physiological stress that requires high energy and significant protein synthesis for spine growth and function. It also involves the translation of mRNAs bearing multiple uAUGs and molecular adaptations to support synaptic remodeling, all of which contribute to memory and learning processes [6], [8].

The kinases capable of phosphorylating eIF2α and thus involved in the integrated stress response (ISR), are: i) the double-stranded RNA-activated protein kinase (PKR), activated by viral infection [7]; ii) the PKR-like endoplasmic reticulum kinase (PERK), triggered by endoplasmic reticulum stress [9]; iii) the general control nonderepressible-2 kinase (GCN2), activated by amino acid deficiency [10], [11]; and iv) the heme-regulated inhibitor (HRI), stimulated by heme deficiency, heavy metals [12], and nitric oxide [6], [8]. A precise ISR is especially critical in neurodegenerative diseases, where neurons must adapt to and survive multiple stressors [13]. For instance, *BACE1* upregulation via this mechanism is linked to amyloidogenesis in Alzheimer’s disease (AD) [6], [14]. Therefore, predicting potential mRNA targets of phosphorylated eIF2α could lead to the identification of new candidates for treating neurological diseases.

In this work, we developed a Python script that identifies specific features of a given 5′UTR, and predicts its predisposition to undergo translation upon eIF2α phosphorylation. We focused on identifying new ISR targets and their associated functions, along with the *in vitro* validation of one of these targets.

## 2 Materials and methods

### 2.1 5′UTR database

To obtain a reliable human 5′UTR database, we considered the Ensembl canonical transcript for each human gene. The human gene set from genome build version GRCh38.p14 was retrieved from Ensembl [15]. Unique Gene codes in Ensembl format were extracted and compiled into a list of 19,099 human genes. Separately, *Mus musculus* Ensembl gene IDs were compiled from training and testing datasets. These Ensembl IDs were used to query the Ensembl database using the REST API [16] and extract the canonical transcripts corresponding to each gene. Ensembl provides 5′UTR sequences on its website for each transcript. However, to facilitate faster programmatic access through the API we retrieved these regions differently. Each transcript’s coding sequence and complementary DNA were downloaded using the API. Although explicit 5′UTR are only manually retrievable, they are implicitly available in the API by being encoded in the difference between the 5′ ends of complementary DNA and coding sequences. Therefore, the 5′UTR region was obtained by identifying the 5′ portion of the cDNA not included in the coding sequence. Ensembl IDs were translated into gene symbols using the EnsDb.Hsapiens.v86 R package [17] and g:Profiler [18]. Custom analysis scripts were implemented in Python 3.11.3 [19].

### 2.2 Database filtering for model training and testing

To obtain reliable model training, we used published databases of p-eIF2α-dependent translation, one for training [20] and another for testing [5]. In both of them, the authors used the phosphorylation-deficient mutant *Eif2s1* S51A knock-in, and assay ribosome-bound mRNA.

For the training database, we filtered the raw data from RNA-seq and Ribo-seq experiments from Kalish et al. [20]. In their experiments, authors used maternal immune activation in E18.5 mice. They observed changes in the protein translation dependent on eIF2α phosphorylation in male mice, as validated by immunoblotting and the knock-in model. Consequently, we only used data from male mice. First, we selected the targets with no major changes in total RNA content (RNA-seq: −0.2 < log2FC < 0.2) and significant changes in ribosome-bound RNA (Ribo-seq: log2FC < −0.2 or log2FC > 0.2, with a false discovery rate (FDR) < 0.05). Then, we classified them as positives or negatives depending on their Ribo-seq log2FC value, defining as positives those up-regulated under stress conditions and vice versa.

For testing the database, we filtered the raw data from RNA-seq and Ribo-seq experiments performed by Amiri et al. [5] based on translation efficiency. To obtain this value, they divided the RPKM value of Ribo-seq results against the one from RNA-seq. First, we selected statistically significant targets for the translation efficiency (padj < 0.05). Then, we grouped them by fold-change (FC). On one hand, if the translation efficiency log2FC (eIF2α^A/A^ compared with eIF2α^+/+^) < -0.2, we considered them positive, meaning their translation would be enhanced in a p-eIF2α activation scenario. On the other hand, if the translation efficiency log2FC (eIF2α^A/A^ compared with eIF2α^+/+^) > 0.2, we considered them negative, meaning their translation would be reduced in a p-eIF2α activation scenario.

As a final filtering step, we removed from the training dataset, since it contained more entries, the transcripts that were repeated in both databases. There were 39 repeated transcripts in total, 10 positives and 29 negatives. In all instances, positives and negative cases coincided in the two databases (Chi-square test versus random distribution, p-value = 4.23806E-10), providing an extra control of the input databases.

Finally, we obtained 321 positive and 418 negative transcripts for the training dataset (Table S1), and 143 positive and 244 negative transcripts for the testing dataset (Table S2).

### 2.3 Training

The model was based on an MLR using four variables from the 5′UTRs to predict a score: length, GC percentage, uORFs, and similarity to the Atf4 positive control (Atf4like). Moreover, regardless of the score, we only considered positive targets containing at least one uORF, allowing the previously described biological mechanism for p-eIF2α-dependent translation to occur. Before performing the MLR analysis, we filtered the training dataset to include only transcripts with at least one uORF.

For the MLR model, we obtained a value for each beta coefficient using the following formula:

$$\mathrm{Score}=\frac{\exp\left(\mathrm{Prescore}\right)\;}{1+\exp\left(\mathrm{Prescore}\right)\;}$$

Prescore=β0+β1*A+β2*B+β3*C+β4*D+β5*A*B+β6*A*C+β7*A*D+β8*B*C+β9*B*D+β10*C*D

Where: A = uORFs, B = Atf4like, C = Length, D = %GC.

The first step to train the model was to obtain the A, B, C and D values for each 5′UTR in order to be able to obtain beta values.

- A (uORFs) is a discrete variable that counts all possible uORFs starting by AUG. To achieve this, we considered each of the three possible reading frames and counted the AUGs with an “adequate” Kozak consensus sequence within them [21]. We excluded those too close to the previous one (less than 30 nucleotides apart, considering a minimum space for the ribosome to leaky-scan the first uAUG and try to translate a second uAUG [22]) or located within an already opened reading frame from a previous uORF lacking a STOP codon.

- B (Atf4like) is a categorical variable (0, 1, 2, or 3) that counts the reading frames in which we found a uORF that has not yet encountered a STOP codon and was preceded by at least one already terminated uORF (it was not necessary for the already terminated uORF to be in the same reading frame).

- C (Length) is a continuous variable that counts the number of nucleotides present in the 5′UTR.

- D (%GC) is a continuous variable that represents the percentage of G or C nucleotides relative to the total length.

Once we have the input variables for each 5′UTR, we split the filtered training dataset into four subsets, each containing a ratio of positive to negative targets that was as close to equal as possible. Then, we clustered the subsets in the four possible groups of three, in order to use these clusters to train the model. For each cluster, a MLR model was trained using sklearn.linear_model.LogisticRegression module from Python 3.11.3 [23]. We tested each MLR model using the remaining subset as the test set. Sensitivity, specificity and positive predictive value (PPV) for each score threshold from 0 to 0.95 with a 0.05 interval are plotted in Fig. S1A.

To obtain the final model, we averaged for each coefficient from the four sub-models. Then, we validated its performance by plotting its sensitivity, specificity, PPV and likelihood ratio for each score (Fig. S1B). The final code of the model was written in File. S1.

### 2.4 Testing and running

To validate the reproducibility of the model *in silico*, we assayed the transcripts from the testing database and plotted the statistics (Fig. S1C). As we aim to predict reliable targets for p-eIF2α-driven translation, we prioritized a high specificity value over the sensitivity. Therefore, we selected 0.7 as the threshold, as it yielded the highest likelihood ratio. We used Fisher′s exact test for statistical analysis of the model (Table S3). We defined positive hits as those with both a score higher than 0.7 and at least one uORF. After testing, we ran the code on the 5′UTRs from the human transcriptome. We summarized the hits along with their length, %GC, uORFs and scores in Table S4.

Furthermore, to validate the uORF scanning capability of our algorithm, we compared transcripts in which we detected at least one uORF with predicted AUG-initiated uORFs from uORFdb (Unified uORF Database). This resource combines experimental evidence and computational predictions for uORF annotation [24]. A high proportion of our predicted candidates were present in the database (Chi-square p-value < 0.0001, Odds Ratio = 23.94467887, Yule’s Q = 0.91982258).

### 2.5 Gene Ontology (GO) enrichment analysis

We employed GO enrichment analysis [25], [26] to identify the most prevalent differentially represented biological processes, molecular functions, and cellular components [27] among our hits. We sorted the terms with an FDR lower than 0.05 in descending order of fold enrichment.

### 2.6 Interactome

The list of hits contained 473 genes. We constructed a protein-protein interaction (PPI) network using these genes with STRING-db [28], selecting *Homo sapiens* as the organism. We downloaded the functional enrichment of these and sorted them from lowest to highest FDR. We specifically examined the top 20 terms for their relevance to related biological processes. We also extracted the network and visualized it in Cytoscape [29]. In order to categorize these terms according to which cellular signaling pathways or functions were more dependent on these proteins in health (e.g., long term potentiation or synaptic plasticity in brain) versus pathology (e.g., AD), we carried out a literature review. Therefore, the categorization of the enrichments was based on whether they were more frequently related to normal or disease state in literature.

### 2.7 Cloning of SLC30A4 5′-untranslated region

To further validate the reproducibility of the model *in vitro*, we extracted total RNA from the human neuroblastoma cell line SH-SY5Y and carried out one-step RT-PCR using a kit (Qiagen) with custom-designed primers to amplify the 5′UTR from our hit *SLC30A4*: 5′-AGACCTGGGGAGCCGGCCTCCA-3′, 5′-AGCCGGCCATGGCAGAGGCTGA-3′. We isolated and purified the PCR product, a single band matching the molecular weight of the *SLC30A4* 5′UTR (∼270 nt), from an agarose gel using the Illustra™ GFX™ PCR DNA and Gel Band Purification Kit (GE Healthcare). We then inserted the 5′UTR DNA fragment into a modified pGL4.10[luc2] vector from Promega containing the SV40 promoter, between *Kpn*I and *Hin*dIII restriction enzyme sites.

### 2.8 Transient DNA transfection of HEK-293 cells and Luciferase assay

HEK-293 cells were used for the luciferase assays because of their well-documented high transfection efficiency in transient expression systems, ensuring the reliability and reproducibility of the assays [30], [31]. We seeded HEK-293 cells in 96-well plates at a density of 12,000 cells per well, and grew them for 24 h with Dulbecco's Modified Eagle Medium plus 10 % fetal bovine serum and 1 % penicillin/streptomycin. Afterwards, we transfected a total of 100 ng of DNA into each well using Lipofectamine 3000 transfection reagent (Thermo Fisher), adjusting to the following conditions: 100 ng of pcDNA3 plasmid as blanks, and 25 ng of Renilla + 25 ng of pGL4.10 construct and 25 ng of Renilla + 25 ng of pGL4.10–5′UTR·*SLC30A4* construct as test samples. After 24 h, we incubated the corresponding wells with 100 μM salubrinal (Sigma) for 3 h. Firefly and Renilla luciferase activities were assayed using the Dual-Glo™ Luciferase Assay System (Promega) following the manufacturer's instructions. Luminescence was measured using a VICTOR Nivo Multimode Plate Reader (PerkinElmer). Statistical analyses were performed using an unpaired Student’s *t*-test.

## 3 Results

### 3.1 Gene Ontologies

All significant GO terms for biological processes, cellular components and molecular functions are shown in Fig. S2, and classified as specified in Tables S8-S10.

**Fig. 2:**
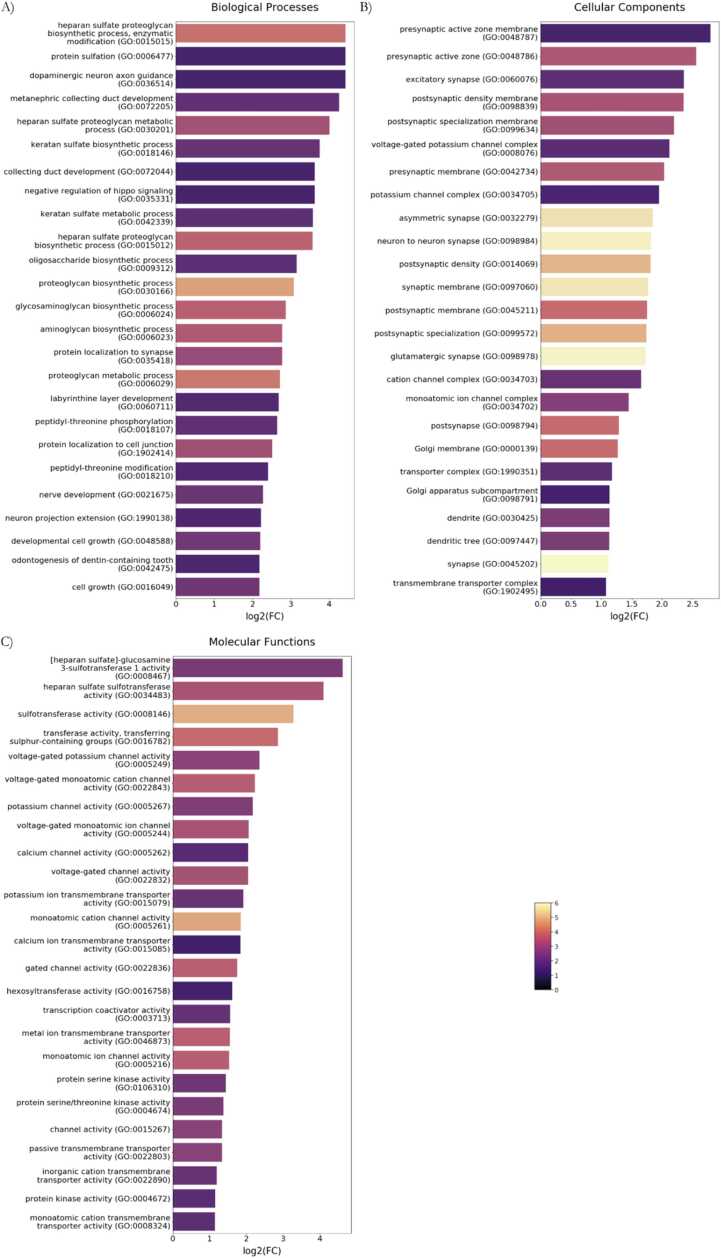
GO term analysis of all positive genes. Bar charts showing the top 25 GO terms for A) biological process, B) cellular component, and C) molecular function, ranked by log2 (fold enrichment). The magma color scale represents the -log10 (FDR).

We found several hits at the biological processes level. Among the processes positively enriched, we highlight those related to neuronal and synaptic growth. More specifically, focusing on neural development and synaptoplasticity we found dopaminergic neuron axon guidance (GO:0036514), protein localization to synapse (GO:0035418), nerve development (GO:0021675) and neuron projection extension (GO:1990138) (Fig. 2A). Other interesting hits regarding organ development and morphogenesis involved development of the collecting duct, the labyrinthine layer from the placenta and odontogenesis, together with the negative regulation of hippo signaling (GO:0035331), a signaling pathway that controls organ size and tissue homeostasis by regulating cell proliferation, apoptosis and stem cell maintenance. Overall, across all significant ontologies, we found an overrepresentation of functions related to cell polarity and morphogenesis, ion transport, protein modification and signaling, and metabolism. As expected under stress conditions, pathways involved in protein synthesis were clearly underrepresented (Fig. S2A). Finally, regarding the specific genes, the most represented ones within the top 25 were: *HS3ST5*, *HS3ST2*, *HS3ST3A1*, *HS6ST1*, *HS6ST3*, *UST*, *CHST5*, *STGAL2*, *DSE* and *STK11* (Table S5). This highlights a marked overrepresentation of heparan sulfate proteins, A group of proteins that function in signaling, neuronal development, synaptic plasticity, and synaptic maintenance.

Regarding the cellular components GO terms with a significant FC, we found a positive enrichment of synaptic structures and ion channels (Fig. S2B). Among the top 25 GO terms ordered by fold-enrichment (Fig. 2B), the most represented genes encoded proteins involved in synaptic structure and function, including N-methyl-D-aspartate receptors (NMDARs, *GRIN2A*, *GRIN2D*), sodium (*SCN8A*) and potassium (*KCNC2*, *KCNC4*, *KCNJ8*) channels, scaffolding proteins (*SHANK1*), and signaling molecules (*LRRC4*, *PRR7*, *ABHD17A*, *NETO1*, *LRRN2*) (Table S6). Their interactions and regulatory roles are essential for maintaining synaptic integrity, signaling and plasticity.

Finally, regarding the significant molecular functions, there was a positive enrichment in Ionic Channels, Transferase, and Kinase activity (Fig. S2C). The overrepresentation of both potassium and calcium gating ion channels was observed in the top 25 GO terms ordered by fold-enrichment (Fig. 2C), with the appearance of voltage-gated potassium channel activity (GO:0005249), voltage-gated monoatomic cation channel activity (GO:0022843), potassium channel activity (GO:0005267), voltage-gated monoatomic ion channel activity (GO:0005244), calcium channel activity (GO:0005262), voltage-gated channel activity (GO:0022832), and metal ion transmembrane transporter activity (GO:0046873). Some of the most represented genes within those GO terms were potassium channels *HCN4*, *KCNN1*, *KCNH3*, *KCNK12*, *KCNQ3*, *KCNJ8*, *KCNQ4*, *KCNJ12*, *KCNB2*, *KCNK2*, *KCNC2*, *KCNC4* and *TRPM5*. Regarding calcium ion transport, we highlight the presence of *CACNA1A*, *GRIN2A*, *GRIN2D*, *TRPC1*, *TRPC3* and *PIEZO2*. Interestingly, within some of those molecular functions, we find genes encoding for proteins that specifically regulate other cations homeostasis, such as the zinc transporters *SLC30A4* and *SLC39A3* (Table S7).

### 3.2 Interactome

**Fig. 3:**
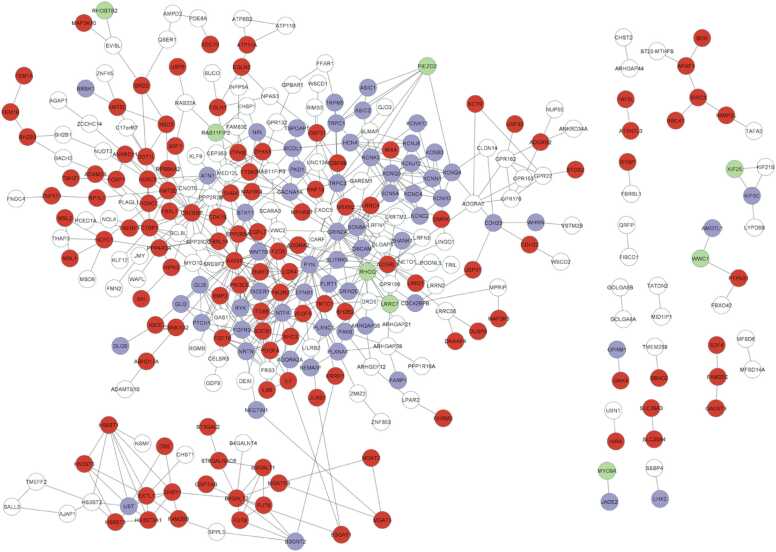
Interactome network. Synaptoplasticity-related proteins are depicted in green, disease-related proteins in red, and proteins related to both groups in purple. Proteins that are not related to any of these pathways are removed from the network for visualization purposes.

We next performed an interactome analysis to evaluate the processes and common proteins involved in health and disease. The resulting network contains 459 nodes and 533 edges (Fig. 3), with a PPI enrichment p-value < 1.0E-16. According to the literature, we highlighted synaptoplasticity-related health processes (green in Fig. 3) as follows: voltage-gated cation channel activity (GO:0022843), voltage-gated ion channel activity (GO:0005244), cation channel activity (GO:0005261), neuron projection morphogenesis (GO:0048812), neuron projection development (GO:0031175), and establishment or maintenance of cell polarity (GO:0007163). On the other hand, we defined the following as disease-related processes (red in Fig. 3): metal ion transmembrane transporter activity (GO:0046873), nervous system development (GO:0007399), protein modification process (GO:0036211), multicellular organism development (GO:0007275), and glycoprotein metabolic process (GO:0009100). Proteins participating in both groups are depicted in purple (Fig. 3).

### 3.3 SLC30A4 in vitro validation

**Fig. 4:**
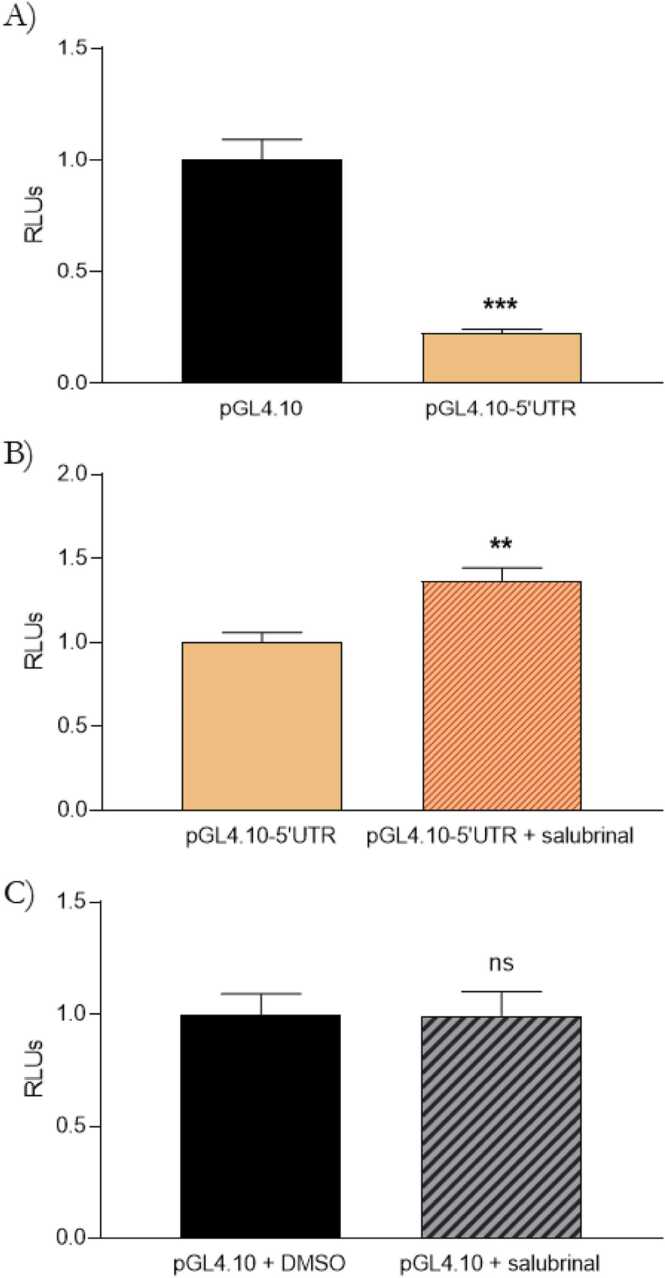
*SLC30A4* translation is induced by ISR. Quantification of the relative changes in relative light units (RLUs) from HEK-293 cells. A) Cells were transfected with pGL4.10 or pGL4.10–5′UTR·*SLC30A4.* B) pGL4.10–5′UTR·*SLC30A4* transfected cells were treated with DMSO or 100 µM salubrinal for 3 h. C) Cells were transfected with pGL4.10 and treated with DMSO or 100 µM salubrinal for 3 h. Data are the mean ± SEM of three independent experiments performed in quintuplicate. **p < 0.01, ***p < 0.001 versus the respective controls by unpaired two-tailed Student′s *t* test analysis.

To further validate the reliability of our predicted targets and explore a novel ISR effector molecular pathway, we cloned *SLC30A4* 5′UTR upstream of a luciferase reporter gene in the HEK-293 cell line. First, we confirmed its inhibitory effect over translation in the absence of ISR activation (Fig. 4A). Moreover, we challenged the pGL4.10–5′UTR·*SLC30A4*-transfected cells with salubrinal, an inhibitor of eIF2α dephosphorylation that induces a sustained ISR state (Fig. 4B). As expected, we observed an increase in the luciferase reporter signal, confirming the specific involvement of ISR activation in *SLC30A4* translation. Salubrinal alone did not affect luciferase expression (Fig. 4C).

## 4 Discussion

Although the datasets used to construct the model [5], [20] were derived from fetal mouse brain samples, they were selected for their comprehensiveness in terms of the number of ISR-responsive proteins identified. Nevertheless, the model provides a valuable framework for identifying potential ISR-regulated proteins across diverse research fields and developmental stages, including human development, adulthood, and senescence. To support this, we have developed an online tool, *StressTranslatome*, available at https://sbi.upf.edu/StressTranslatome, which enables the prediction of proteins activated by the ISR.

### 4.1 Physiological role of p-eIF2α-dependent proteins and their implication in synaptoplasticity

The ISR is a complex signaling mechanism triggered by various physiological and pathological stimuli that help the cell adapt and survive under stressful conditions. The ISR is based on eIF2α phosphorylation, which enhances the translation of mRNAs coding for stress-associated proteins. These mRNAs are sequestered in neurons within stress granules, which are RNA-protein macrocomplexes that protect cellular RNAs from degradation until their translation can be safely restored [32], [33]. During synaptic growth, these granules are utilized to facilitate the rapid production of proteins essential for learning and memory processes. For instance, during early long-term potentiation (LTP), both glutamate [6], [8] and Wnt [34] signaling pathways act as upstream stressors activating HRI, one of the eIF2α kinases. While eIF2α phosphorylation promotes early long-term potentiation (LTP), it hinders late LTP, indicating that precise regulation of its phosphorylation state is crucial for optimal synaptic function and memory formation [35], [36].

The model proposes several downstream effectors of eIF2α phosphorylation implicated in neuronal function and development. Our findings underscore the importance of voltage-gated ion (GO:0022843, GO:0005244) and cation channels (GO:0005261), and neuron growth and morphogenesis (GO:0048812) in maintaining synaptic plasticity [37]. Moreover, calcium influx through synaptic NMDARs, such as *GRIN2A* and *GRIN2D*, modulates LTP and long-term depression [38]. Furthermore, homeostatic plasticity mechanisms stabilize neuronal and circuit activity [39], [40], such as actin modelling through Rho GTPases (*RHOQ*) [41], [42].

Moreover, synaptic structure and ion transport disruption caused by some of the identified hits are associated with several neuropathies. For instance, *SHANK1* plays a key role in associative learning and excitatory synaptic transmission [43], [44]. Its haploinsufficiency due to either deletions or mutations has been associated with autism spectrum disorders [45], [46]. *LRRC4* regulates the formation of excitatory synapses and promotes axon differentiation, and it has been reported to promote a neuroprotective effect in experimental autoimmune encephalomyelitis [47]. Moreover, its microdeletion, modeled by a knock-out mouse, leads to autistic-like behaviors responsive to NMDAR modulation [48]. *NETO1* guides development of glutamatergic connectivity in the hippocampus by regulating axonal growth [49], and variations of the gene have been associated with schizophrenia [50]. Finally, *ABHD17A* has depalmitoylating activity toward DLG4/PSD95 and N-Ras, which influences synapse stability and signaling [51].

### 4.2 Pathophysiological role of p-eIF2α-dependent proteins and their implication in neurodegeneration

The ISR pathway has been linked to several neurological diseases [13]. Dysregulation of this pathway contributes to neurodevelopmental disorders such as fragile X syndrome [52], Down syndrome [53], and leukoencephalopathy with vanishing white matter disease [54], and susceptibility to AD [55]. It is also known that protein misfolding, a hallmark of many neurodegenerative diseases, triggers reticular stress, leading to PERK-mediated eIF2α phosphorylation [13].

However, the role of ISR activation in neurodegenerative diseases is complex and context-dependent. For example, the inhibition of eIF2α dephosphorylation shows neuroprotective effects in α-synuclein transgenic Parkinson’s disease (PD) mice [56] and Charcot-Marie-Tooth mutant mice [57]. Conversely, it exacerbates disease in a prion-related mouse model [58]. Similarly, Guanabenz (another eIF2α dephosphorylation inhibitor) delays disease progression in some amyotrophic lateral sclerosis (ALS) models [59], [60], but worsens it in others [61]. In AD, CCT020312 (a PERK activator) produces neuroprotection [62], while a PERK inhibitor, GSK2606414, also shows protective effects [63]. These puzzling results highlight the need for a precise understanding of ISR pathway modulation and its outputs.

Focusing on AD, our results suggest that eIF2α-ISR pathway influences several processes implicated in the disease’s etiopathology. First, it influences metal ion transmembrane transporters activity (GO:0046873), which could lead to an enhancement of the amyloid β-peptide (Aβ) aggregation pathway through the formation of metal complexes [64]. Furthermore, regulation of redox-active metal ions has been proposed as a strategy to prevent AD [65]. For instance, *KCNC4*, which encodes for voltage-dependent potassium channel K_V_3.4, is overexpressed in AD due to Aβ deposition, contributing to apoptosis [66], [67]. Second, neural plasticity is severely affected in AD, leading to cognitive deterioration and loss of learning and memory [68]. Finally, protein modification processes influence AD pathology. Heparan sulphate proteoglycans, which highlights as the most overrepresented biological process, has been found to be elevated in the cerebrovasculature of AD patients with severe cerebral amyloid angiopathy [69], and they have been proposed to play a role in tau protein aggregation to form the neurofibrillary tangles, a hallmark of AD [70]. Moreover, we also find among the identified targets the tau kinases *BRSK1*, *TTBK1* and *FYN*, which regulate tau accumulation through its phosphorylation [71], [72], [73], potentially leading to tau intracellular aggregation.

Besides AD, *GRIN2A* and *GRIN2D* are NMDARs, whose associated encephalopathies range from intellectual disability to epilepsy-aphasia spectrum phenotypes [74]. *PRR7* acts as a synapse-to-nucleus messenger to promote NMDAR-mediated excitotoxicity in neurons in a JUN-dependent manner [75].

Moreover, the cationic channel hits resulting from our analyses (GO:0005261) may underlay pathological processes. Thus, some voltage-gated sodium channel *SCN8A* variants are related to epilepsy-related phenotypes [76]. *KCNJ8* is expressed in microglia and modulates neuroinflammatory response. Its deficiency dramatically exacerbated dopaminergic neuron death accompanied by microglia activation in a mouse model of PD [77]. Some variants of *KCNQ3* that alter channel function have been linked to some rare forms of epilepsy [78]. *CACNA1A* encodes for Ca_V_2.1, a voltage-dependent calcium channel. Both gain and loss of function mutations in the gene are linked to a group of diseases associated with neuronal calcium disbalance, including hemiplegic migraine or congenital ataxia [79]. Finally, *TRPC3* is a calcium channel highly expressed in the hippocampus that contributes to seizure-induced neuronal cell death [80].

### 4.3 Zinc transportation: a novel effector in ISR

Examination of the interactome in conjunction with the identified database hits has enabled us to underscore the significance of lesser-known mechanisms, such as zinc transportation, in the context of ISR. Zinc is an essential micronutrient that performs various structural, catalytic and regulatory functions in the human body. Its systemic and cellular homeostasis is tightly regulated by zinc transporters [81], [82]. Among the multiple regulatory functions of zinc, in a neuronal context, it acts as a synaptic transmission modulator. It is released from presynaptic neurons upon excitation, and binds to and influences the activity of various channels from postsynaptic neurons [81]. However, both the deficiency and excess of zinc have been associated with neurological conditions, such as depression, schizophrenia, AD, PD, and ALS [83]. Therefore, given the critical role of zinc homeostasis in neuronal pathophysiology, together with the predicted importance of cation exchange in ISR and synaptoplasticity, we decided to perform *in vitro* validation of *SLC30A4*. This transcript, together with SLC39A3, encodes for zinc transporters (zinc transporter 4 (ZnT4) and ZIP3, respectively), and both have already been associated with neurodegeneration processes [84], [85] and AD [86], [87]. ZnT4 protein expression is known to increase in preclinical, early, and late AD patient samples [87], [88]. As ZnT4 exports zinc from the cytosol into the lysosomal and trans-Golgi compartments, it has been hypothesized that its upregulation would allow excess zinc to be removed from the cytoplasm before it can cause harm in the context of neurodegeneration [89], [90]. Our findings identify a novel mechanistic connection between zinc homeostasis regulation and ISR activation in neurodegenerative diseases.

The successful validation of SLC30A4 serves as experimental *in vitro* proof that the model's predictions are functional. This approach provides a reliable framework for validating additional targets in future studies, overcoming inherent *in silico* limitations while significantly enhancing the robustness of our computational model.

## 5 Conclusions

Our predictive model identifies p-eIF2α-dependent translation targets, with particular relevance to synaptoplasticity and neurodegeneration-related pathways. This framework not only reveals novel molecular players in the integrated stress response (ISR), but also establishes a foundation for the systematic discovery of therapeutic targets in neurodegenerative diseases.

## CRediT authorship contribution statement

**Víctor Herrera-Fernández:** Writing, Visualization, Validation, Software, Methodology, Investigation, Formal analysis, Data curation, Conceptualization. **Hugo Fanlo-Ucar:** Methodology, Investigation, Formal analysis. **Patrick Gohl:** Validation, Software, Methodology, Investigation, Formal analysis, Data curation, Conceptualization. **Melisa Ece Zeylan:** Validation, Software, Methodology, Investigation, Formal analysis, Data curation, Conceptualization. **Simge Senyuzc:** Methodology, Investigation, Formal analysis. **Ozlem Keskin:** Validation, Software, Methodology, Investigation, Formal analysis, Data curation. **Attila Gursoy:** Validation, Software, Methodology, Investigation, Formal analysis, Data curation, Resources. **Rubén Vicente:** Validation, Methodology, Investigation, Formal analysis, Data curation. **Baldo Oliva:** Writing, Visualization, Validation, Software, Methodology, Investigation, Resources, Formal analysis, Data curation, Conceptualization. **Francisco J Muñoz:** Writing, Visualization, Validation, Software, Methodology, Formal analysis, Data curation, Conceptualization, Resources.

## Funding

This work was supported by the Spanish Ministry of Science and Innovation and Agencia Estatal de Investigación plus FEDER Funds through grants PID2023-149767OB-I00 funded by MICIU/AEI/10.13039/501100011033 and by 'ERDF A way of making Europe' (FJM), PID2023-150068OB-I00 funded by MICIU/AEI/10.13039/501100011033 and by ‘ERDF A way of making Europe’ (BO) and PID2022-136511OB-I00 (RV). This work was also funded by the Spanish Institute of Health Carlos III by project reference AC20/00009 -FEDER/UE and ERANET ERA-CVD_JTC2020-015; and, ‘Unidad de Excelencia María de Maeztu’ CEX2024-001431-M, funded by MICIU/AEI/10.13039/501100011033. This project was funded in part by TUBITAK Research GrantNo: 220N252 (AG).

## Declaration of interest

None.

## Data Availability

Supplementary data to this article (Supplementary figures and tables) can be found on line at https://doi.org/10.1016/j.csbj.2025.07.015. The predictor code for the analysis performed in this article (File S1) is available at GitHub (https://github.com/structuralbioinformatics/EiF2alpha-Predictors) and Zenodo (DOI: 10.5281/zenodo.139142139).
