# Supplementary material for: Predicting cellular adaptation proteins dependent on eIF2α regulation under stress conditions: Physiological and pathophysiological implications in neuronal function
Source: Comput Struct Biotechnol J. 2025 Jul 12;27:3127–36. doi: 10.1016/j.csbj.2025.07.015 (PMC12303066; doi:10.1016/j.csbj.2025.07.015)
Supplement: Supplementary file 1 — Supplementary material [file mmc1.docx]

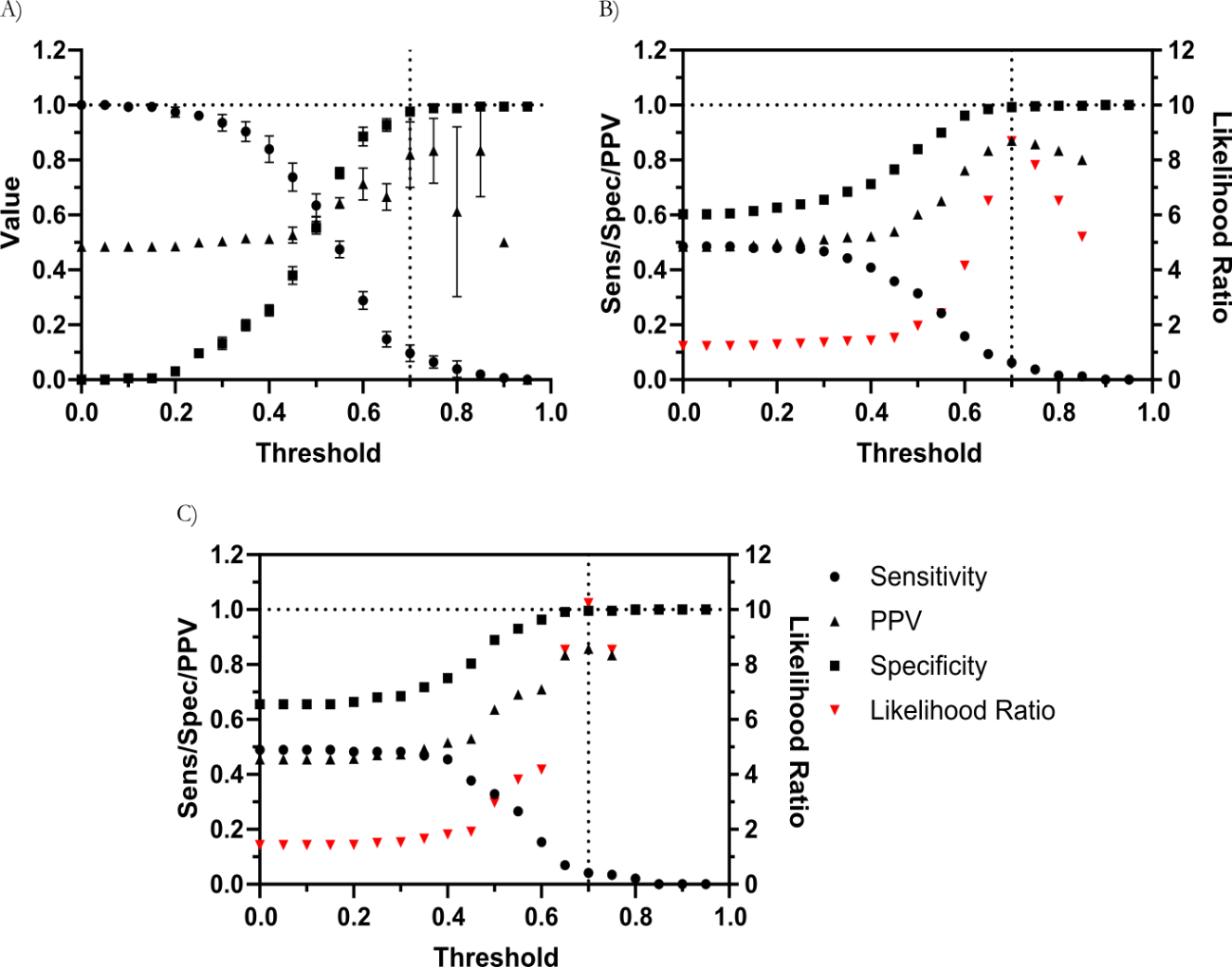


**Fig. S1. Model statistical analysis**. Sensitivity, specificity and PPVs for each threshold ranging from 0 to 0.95 with an interval of 0.05 for the tested datasets. A) Training subsets tested to each complementary testing dataset using a 4-fold (training N=4). Data are means ± SEM. B) Definitive model obtained and tested using the whole dataset. C) Definitive model obtained with the whole training set and tested in an independent dataset. Likelihood ratios (red inverted triangles) are plotted in the right Y axis in B and C.


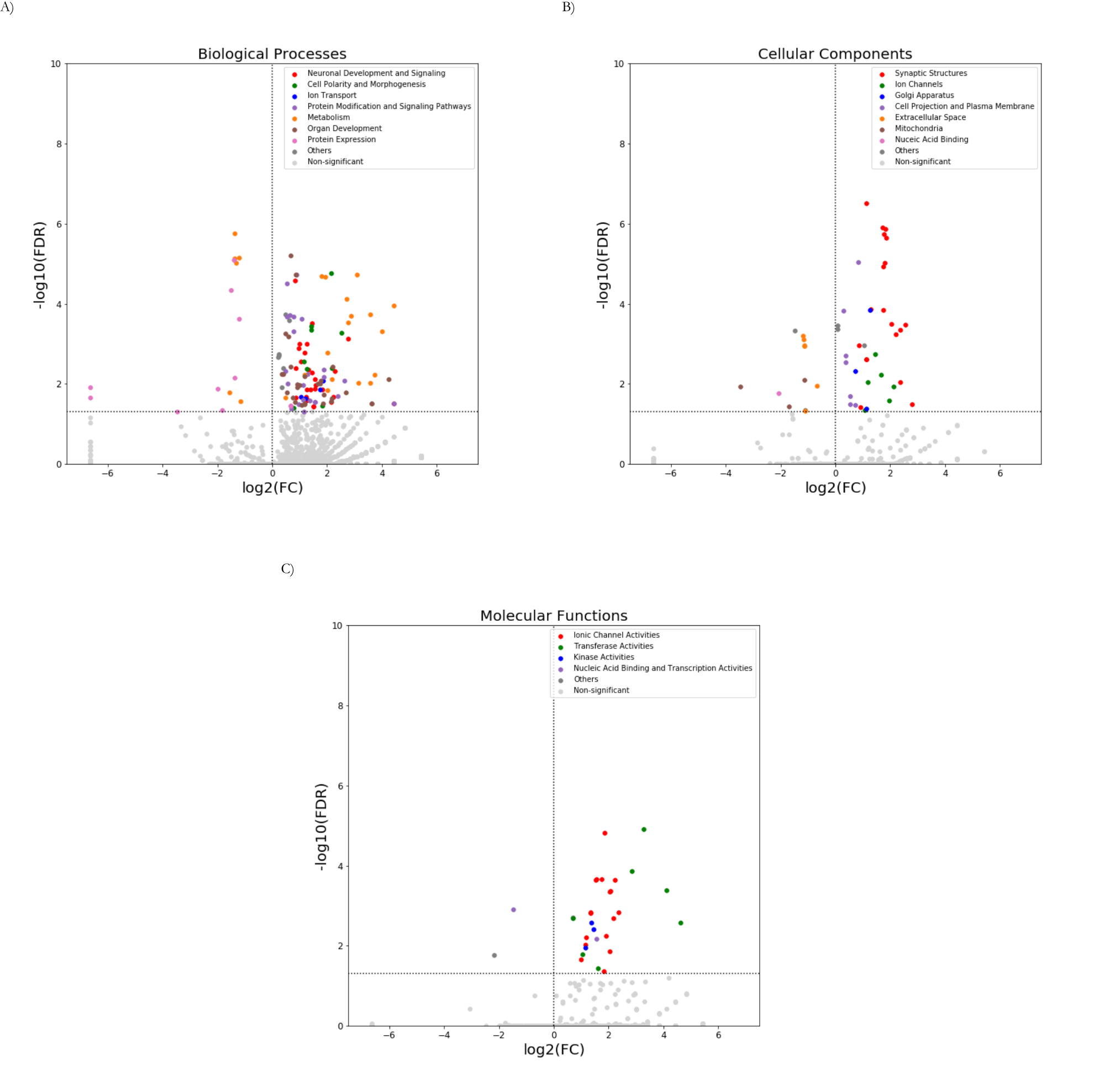


**Fig. S2. Volcano plots from GO enrichment analysis**. GO terms from A) biological processes, B) cellular components, and C) molecular functions enrichment analysis. Intensity is depicted as log2(FC) (X axis) and significance as -log10(FDR) (Y axis). Dots are colored by our GO term classifications.
