# Supplementary material for: Predicting cellular adaptation proteins dependent on eIF2α regulation under stress conditions: Physiological and pathophysiological implications in neuronal function
Source: Comput Struct Biotechnol J. 2025 Jul 12;27:3127–36. doi: 10.1016/j.csbj.2025.07.015 (PMC12303066; doi:10.1016/j.csbj.2025.07.015)
Supplement: Supplementary file 2 — Supplementary material [file mmc2.docx]

**File S1. Python3 code to predict p-eIF2α-dependent translation targets**.

**Table S1. Training dataset**.

**Table S2. Testing dataset**.

**Table S3. Contingency table analysis for model testing**.

**Table S4. List of positive p-eIF2α-dependent traduced targets**. Score and values for each assayed input variable (ORFs, Atf4-like, Length, and %GC) are depicted.

**Table S5. Top 25 biological processes GO terms sorted by log2(FC)**. Rank, log2(FC), -log10(FDR), number of genes and gene symbols are depicted.

**Table S6. Top 25 cellular components GO terms sorted by log2(FC)**. Rank, log2(FC), -log10(FDR), number of genes and gene symbols are depicted.

**Table S7. Top 25 molecular functions GO terms sorted by log2(FC)**. Rank, log2(FC), -log10(FDR), number of genes and gene symbols are depicted.

**Table S8. GO terms clustering used for Supplementary Figure 2A**.

**Table S9. GO terms clustering used for Supplementary Figure 2B**.

**Table S10. GO terms clustering used for Supplementary Figure 2C**.
